# Synthesis of Crosslinkable Alkali-Soluble Resins and Self-Crosslinking Polyacrylic Latexes

**DOI:** 10.3390/molecules30122551

**Published:** 2025-06-11

**Authors:** Min Li, Yansen Wang, Jun Ye, Longhai Guo, Haiqiao Wang

**Affiliations:** 1 State Key Laboratory of Organic-Inorganic Composites, Beijing University of Chemical Technology, Beijing 100029, China; lm2147444318@126.com; 2 Beijing Engineering Research Centre for the Synthesis and Applications of Waterborne Polymers, Beijing University of Chemical Technology, Beijing 100029, China; jsycdfwys@126.com (Y.W.); yejun@buct.edu.cn (J.Y.)

**Keywords:** crosslinkable alkali-soluble resins, self-crosslinking polyacrylate latexes, in situ one-pot method, emulsion polymerization, properties

## Abstract

In the field of water-based inks, the use of alkali-soluble resins (ASRs) as polymeric surfactants for synthesizing polyacrylate latexes has become a mainstream method. This study first designed and prepared crosslinkable ASRs with a diacetone acrylamide (DAAM) crosslinking monomer via emulsion polymerization. These ASRs were then employed as surfactants to synthesize self-crosslinking polyacrylate latexes through an in situ one-pot method, systematically investigating the influence of crosslinkable ASRs on the properties of the corresponding polyacrylate latexes. The research revealed that all prepared polyacrylate latexes exhibited a core–shell structure. With increasing DAAM content in the ASRs, the latex particle size gradually increased while the particle size distribution narrowed. All latexes demonstrated excellent stability, with absolute ζ-potential values exceeding 30 mV. The introduction of DAAM into ASRs significantly increased the glass transition temperature in the high-temperature region of the corresponding latex films, with the tensile strength reaching a maximum of 7.96 MPa. Moderate crosslinking in ASRs substantially improved the water resistance of latex films. Crosslinking degree tests indicated that latex films prepared through either single shell-layer crosslinking or single core-layer crosslinking showed relatively low crosslinking degrees, while only the dual core–shell crosslinking strategy could effectively enhance the film crosslinking degree. However, excessively crosslinked shell layers significantly hindered the crosslinking reaction of DAAM in the core layer, leading to reduced overall film crosslinking. Additionally, incorporating a certain number of DAAM crosslinking groups in ASRs was found to improve the adhesion of corresponding water-based inks on PE and BOPP substrates, with adhesion on BOPP substrates reaching up to 100%.

## 1. Introduction

Driven by the rapid development of the manufacturing sector, the packaging industry has witnessed continuous capacity expansion, while the printing industry has experienced exponential growth. This has directly fueled substantial market demand for ink products. Against this backdrop, the development of environmentally friendly printing materials has become an industry-wide consensus. Water-based inks, owing to their significant ecological advantages, have emerged as a key focus area for advancement [1,2,3].

Water-based inks typically consist of several core components: polyacrylate latex binders, pigments, and functional additives (e.g., wetting agents, defoamers, etc.) [4,5]. As the critical film-forming component in water-based inks, polyacrylate latex binders not only govern the system’s rheological properties, viscosity regulation, and drying kinetics but also directly determine the quality of ink film formation, printability, and interfacial adhesion strength with substrates [6]. Therefore, refining the binder system through molecular design and formulation optimization strategies can effectively overcome key technical challenges in water-based inks, such as water resistance, drying efficiency, and substrate adhesion.

Waterborne polyacrylate latexes have emerged as a pivotal research direction for eco-friendly ink binders due to their exceptional properties, including weather resistance, high transparency, chemical corrosion resistance, and mechanical strength [7,8,9,10,11,12,13,14]. Recent studies have focused on advancing crosslinking modification techniques for these latexes. For instance, Pi et al. [15] developed a low-temperature crosslinking strategy to synthesize polyacrylate latex, significantly enhancing adhesion to polyethylene substrates while maintaining low water absorption. Wen’s team [16] designed a room-temperature multi-crosslinking system that synergistically improved the drying and ethanol scrub resistance on PVC substrates. Wang et al. [17] demonstrated that incorporating isobornyl acrylate (IBOA) monomers into binders effectively modulates latex surface tension, enhancing ink wettability on low-surface-energy substrates (e.g., BOPP/PE), while simultaneous integration of the DAAM/ADH crosslinking system [18] notably boosted ink adhesion. In crosslinking mechanism research, Lee et al. [19] revealed the correlation between crosslinking density and polymerization kinetics via seeded emulsion polymerization, with follow-up work [20] showing that interlayer crosslinking in core–shell structures concurrently optimizes latex stability and film performance. Pedraza et al. [21] confirmed that increasing functional group density on latex particle surfaces enhances film mechanical properties, while Bas and Soucek [22] investigated the impact of different crosslinkers on core–shell latex behavior. Notably, the DAAM/ADH system [23,24,25] stands out for its efficient crosslinking, offering unique advantages in improving overall latex performance. However, polyacrylate latexes prepared with small-molecule emulsifiers face inherent limitations, as emulsifier migration during film formation compromises coating adhesion and increases water sensitivity [26,27,28,29,30,31], potentially exacerbating environmental pollutant accumulation. This has spurred interest in alkali-soluble resins (ASRs) as surfactants for polyacrylate latex synthesis, where ASRs stabilize colloids through synergistic anionic charges (from carboxylate ionization, pKa ≈ 4.5) and steric hindrance [32,33,34]. ASRs offer dual advantages: they are more compatible with conventional radical polymerization than block copolymers, and when used as dispersants, they improve rheology control and pigment-loading capacity, demonstrating clear advantages for industrial production.

ASRs suitable for use as surfactants typically have molecular weights ranging between 5000 and 20,000 Da. Currently, the primary methods for synthesizing ASRs include bulk polymerization and solution polymerization. Industrial production predominantly employs bulk polymerization, while solution polymerization is more commonly used in laboratory settings [35], as this method offers greater control over molecular weight compared to emulsion polymerization. However, the solution polymerization process is less environmentally friendly, may leave solvent residues in the final product, and tends to yield ASRs with relatively broad molecular weight distributions, consequently resulting in lower-quality polyacrylate latexes.

Although ASR-based polyacrylate latexes offer numerous advantages, several critical challenges remain unresolved. Specifically, when synthesizing polyacrylate latexes using ASRs, the ASR content typically needs to reach around 30% or even higher to effectively stabilize micelles. This high ASR requirement leads to two major drawbacks. First, industrially produced ASRs inherently lack crosslinking ability and predominantly reside in the shell layer of latex particles. Their substantial presence in the synthesized latexes severely limits the crosslinking degree of the resulting latex films, resulting in poor resistance to solvents/media and inferior mechanical properties. Second, the thick ASR shell layer surrounding the latex particles creates strong steric hindrance, significantly impeding the crosslinking reaction between DAAM (in the core) and ADH (in the aqueous phase). This further reduces the overall crosslinking degree of the latex films. Therefore, developing effective ASR crosslinking technologies is essential for enhancing film crosslinking degree and improving the performance of latex films and their derivative products.

Theoretically, due to the abundance of carboxyl groups in ASRs, crosslinking agents such as metal ions (e.g., Zn^2+^), aziridine, and carbodiimide—which can react with carboxyl groups—can enhance the crosslinking degree of latex films. However, aziridine and carbodiimide cannot be used in one package and must be added to water-based inks just before application. Inks containing these crosslinkers have a short pot life, failing to meet the demands of modern printing processes for prolonged continuous operation. Meanwhile, metal ion crosslinking results in ink films with poor water resistance, making them unsuitable for practical applications.

**Scheme 1:**
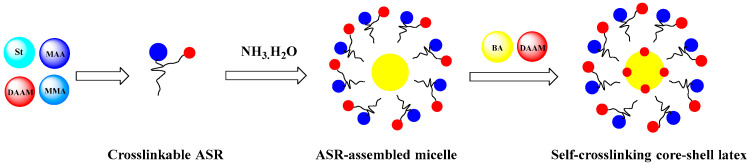
The schematic diagram for the synthesis of the core–shell polyacrylate latexes with varying amounts of DAAM. Blue circles indicate hydrophilic end polymers and red circles indicate DAAM on polymerization.

Herein, one non-crosslinked ASR (control sample) and three crosslinkable ASRs were synthesized via emulsion polymerization using styrene (St), methyl methacrylate (MMA), and methacrylic acid (MAA) along with varying amounts of the DAAM (diacetone acrylamide) crosslinking monomer. Subsequently, these four synthesized ASRs were utilized as surfactants to prepare core–shell structured polyacrylate latexes using a one-pot approach, with the aim of investigating the influence of ASR crosslinking on the properties of corresponding latexes. At the same time, three different crosslinking systems were established: (1) core crosslinking, (2) shell crosslinking, and (3) core–shell dual crosslinking, to examine the contributions of distinct crosslinking strategies to the crosslinking degree of latex films. The specific design is shown in Scheme 1. Finally, the synthesized polyacrylate latexes were applied as binders for the preparation of water-based inks, and the effect of crosslinkable ASRs on the adhesion of corresponding water-based inks on PP and PE film substrates was investigated. To the best of our knowledge, the design concept of preparing crosslinkable ASRs to impart a high degree of crosslinking to the corresponding polyacrylate latex films to improve their water resistance, mechanical properties, etc., has not been reported.

## 2. Results and Discussion

### 2.1. Preparation of Crosslinkable ASRs and Corresponding Self-Crosslinking Polyacrylate Latexes

The molecular weight, molecular weight distribution, and acid value of alkali-soluble resins (ASRs) significantly influence their suitability as surfactants and the performance of the resulting polyacrylate latexes. ASRs suitable for use as surfactants typically have a molecular weight ranging from 5000 to 20,000 Da and an acid value between 130 and 250 mg KOH/g. Therefore, controlling the appropriate molecular weight and acid value is crucial for preparing ideal ASRs.

In this study, ASRs were synthesized via emulsion polymerization using monomers MMA, St, and MAA, along with chain transfer agent dodecyl mercaptan, crosslinking monomer DAAM, and emulsifier SDS (sodium dodecyl sulfate). Four ASRs were prepared with different DAAM contents: ASR0 (0 wt% DAAM), ASR1 (1 wt% DAAM), ASR2 (2 wt% DAAM), and ASR3 (3 wt% DAAM). The theoretical acid value was set at 130 mg KOH/g by adjusting the MAA content, while the molecular weight was controlled at approximately 8500 Da (Mn) by regulating the amount of dodecyl mercaptan.

Subsequently, four core–shell structured polyacrylate latexes—S0, S1, S2, and S3—were synthesized by using ASR0, ASR1, ASR2, and ASR3 as surfactants, respectively, with BA as the core monomer.

Finally, to investigate the effects of two distinct crosslinking modification strategies—core crosslinking versus shell crosslinking—on the crosslinking degree and corresponding properties of latex films, four additional polyacrylate latexes (S0C2, S1C2, S2C2, and S3C2) were designed and synthesized. These were prepared using the aforementioned ASRs as surfactants and BA as the core monomer, with 2 wt% DAAM crosslinking monomer specifically incorporated into the core layer of the latex particles.

### 2.2. Characterization of ASRs

**Figure 1:**
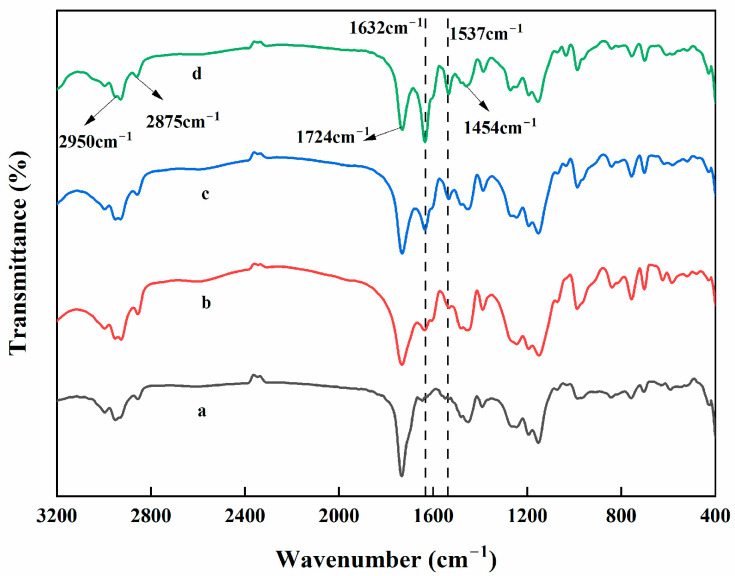
FT-IR spectra of ASRs: (a) ASR0, (b) ASR1, (c) ASR 2, (d) ASR3. The molar amount of ADH added to the ASRs is equal to the molar amount of DAAM.

The four ASR samples synthesized with varying DAAM contents (0 wt%, 1 wt%, 2 wt%, and 3 wt%) were firstly characterized using FT-IR spectroscopy. As shown in Figure 1, in the infrared spectra, distinct vibrational absorption peaks appeared at 2953 cm^−1^, 2862 cm^−1^, and 1454 cm^−1^, which corresponded to the -C-H- vibrations. The absorption peak at 2953 cm^−1^ is usually attributed to the asymmetric C-H stretching vibration, the absorption peak at 2862 cm^−1^ corresponds to the symmetric C-H stretching vibration, and the peak at 1454 cm^−1^ is due to the deformation vibration of the C-H. The presence of these characteristic absorption peaks strongly confirms the presence of hydrocarbon structures in polyacrylate latexes. In addition, the absorption peak of -C=O- in the ester group appeared at 1724 cm^−1^. The ester group is an important structural unit in the acrylate molecule, and the appearance of this absorption peak indicates that the polyacrylate latex contains an ester group structure, which further confirms the successful preparation of the polyacrylate latex. In the alkali-soluble resin with the addition of DAAM and ADH, a deformed vibrational absorption peak of -N-H- at 1537 cm^−1^ was observed. The presence of this peak indicates that DAAM has been successfully introduced into the polymer. DAAM molecules contain amide groups, and the N-H bond in the amide group produces an absorption peak in a specific wave number range. When DAAM is introduced into the polymer, the vibration of its N-H bond shows up as an absorption peak at 1537 cm^−1^ in the infrared spectrum. At the same time, an absorption peak of -C=N- at 1632 cm^−1^ appeared. The presence of this peak indicates that a ketohydrazine crosslinking reaction has taken place and hydrazone has been produced. In the ketohydrazide crosslinking reaction, the keto carbonyl group in DAAM reacts with the hydrazide group in ADH to form a hydrazone structure containing a -C=N- bond. The vibration of this bond is shown in the infrared spectrum as an absorption peak at 1632 cm^−1^. The successful preparation of self-crosslinked alkali-soluble resin was demonstrated by FT-IR spectral analysis.

**Figure 2:**
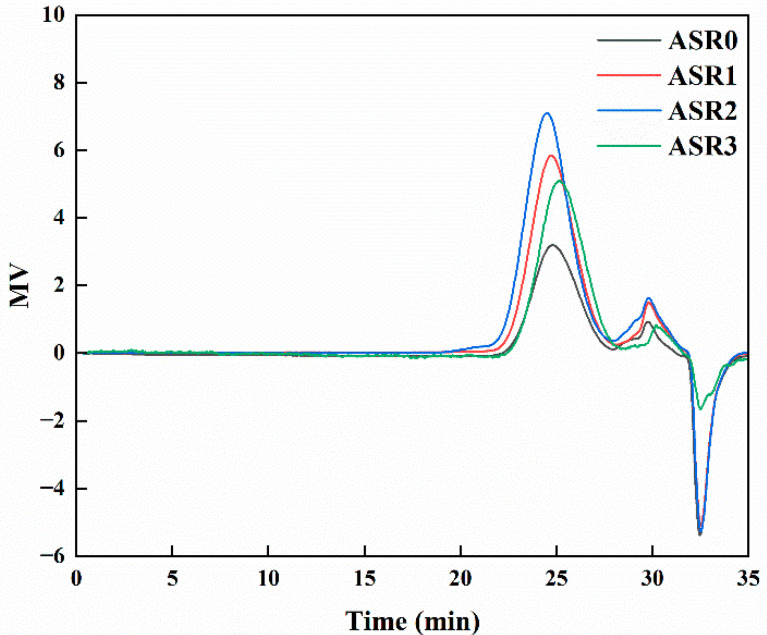
GPC elution profile of the ASRs.

As mentioned above, molecular weight and molecular weight distribution is an important performance parameter to measure the quality of ASR. The molecular weights and their distributions of the obtained ASRs were tested using GPC in the experiments. Figure 2 shows the elution profile curve of ASRs obtained from the test. Table 1 summarizes molecular weights and polydispersity index (PDI) of the molecular weights.

From Figure 2 and Table 1, it can be clearly seen that the average molecular weight of the synthesized ASRs ranges from 8124 to 10,370 Da, which is very close to the famous product J678 from BASF in Germany. As indicated in Table 1, the polydispersity index of ASRs is between 1.55 and 1.78, demonstrating a relatively narrow molecular weight distribution. PDI is an important indicator of the performance of ASRs, and the narrow molecular weight distribution positively affects the properties of ASRs in many ways. In the synthesis of ASR, n-dodecanethiol plays a pivotal role as a chain transfer agent. During synthesis, n-dodecanethiol effectively reacts with the growing polymer chain radicals, transferring the radicals to itself and thus terminating the chain growth. Due to the high chain transfer activity of n-dodecanethiol, it is possible to precisely control the molecular weight of ASRs under appropriate reaction conditions. Also, the presence of n-dodecanethiol promotes the narrowing of the molecular weight distribution. This is due to the fact that the chain transfer reaction occurs relatively uniformly, allowing the resulting polymer chains to be close in length, which ultimately leads to a narrower molecular weight distribution of the ASR.

### 2.3. Latex Particle Size and Size Distribution

**Figure 3:**
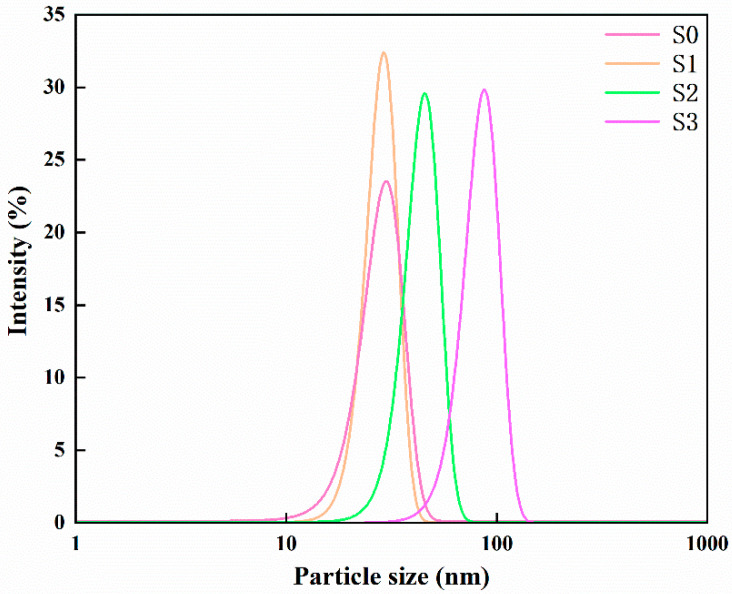
Particle size and particle size distribution of the four polyacrylate latexes synthesized using different ASRs.

The particle size and particle size distribution of the four latexes were first characterized. Figure 3 shows the characterization results.

From Figure 3, it can be seen that the particle sizes of all four latexes showed a unimodal distribution, and the particle sizes were small, located in the range of 60.1~110.3 nm; the particle size distribution interval was 0.20~0.37. The above results indicate that all four ASRs have good surface activity, and they can be used for the preparation of polyacrylate latexes with small particle sizes. Additionally, it can also be found that, with the gradual increase in the amount of DAAM on the ASR, the particle size of polyacrylate latex synthesized by the one-pot method tends to increase gradually, while PDI becomes smaller. When the amount of DAAM in the shell layer increases, its dissolution in water also increases. During emulsion polymerization, this change resulted in a significant increase in the probability of oligomer nucleation. The oligomer nucleation process consumes some of the primary radicals, which in turn leads to a corresponding decrease in micelle nucleation. Nucleation of micelles is one of the important ways to form latex particles in emulsion polymerization, and the reduction in its number directly affects the number of latex particles generated. In emulsion polymerization systems, a reduction in the number of latex particles means that the space occupied by individual latex particles becomes relatively larger, leading to an increase in the size of the latex particles. At the same time, due to the reduced number of latex particles, the range of differences in particle size between them narrows and the particle size distribution becomes narrower.

### 2.4. Latex Fundamental Performance Indicators

The ζ potential, as a key measure of the total charge acquired by the particles in a given medium, is regarded as one of the important benchmarks for determining the stability of colloidal systems. In general, when the absolute value of the ζ potential of a suspension or dispersed system exceeds 30 mV, it means that the system is able to maintain a steady state at the physical level.

As can be seen from Table 2, the absolute values of ζ potentials of the four latexes were S0, 39.0; S1, 41.7; S2, 40.4; and S3, 43.1. Obviously, the polyacrylate latexes synthesized by the one-pot method through the in situ generation of ASRs containing different amounts of DAAM are all above 39 mV. A high absolute zeta-potential value indicates strong interparticle electrostatic repulsion, thereby enhancing latex colloidal stability. Moreover, latexes with DAAM are more stable than those without DAAM. This is because after the addition of DAAM, on the one hand, the PDI of the latex becomes smaller, and the inhomogeneity between particles decreases, which correspondingly reduces the possibility of aggregation and precipitation of particles; on the other hand, the increase in the particle size of the latex particles reduces the total surface energy of all particles in the system, which also correspondingly reduces the possibility of aggregation and precipitation of particles, thus improving the stability of the latex.

Table 2 lists the conversion of the monomers and solid content of the four polyacrylate latexes.

One can find that when the amount of DAAM on the ASR was increased, no significant fluctuating changes in monomer conversion were observed in the synthesis of polyacrylate latexes using it as a surfactant. From the actual performance of the experimental data, the monomer conversion rate is always maintained at a high-level state, basically above 95%. This phenomenon suggests that under the current established experimental environment, feedstock ratios, and reaction conditions, changes in DAAM dosage have a relatively small impact on the monomer conversion rate. Although the monomer conversion rate itself is already at a high level, in order to further purify the emulsion system in depth and maximize the elimination of residual monomers in the system, a post-elimination treatment was introduced in this study. That is, the strong oxidizing agent tert-butyl hydroperoxide and the reducing agent vitamin C are added. Unsurprisingly, after this post-elimination process, the monomer conversion of the latexes, such as S3, is improved by leaps and bounds, reaching about 99.6%.

### 2.5. Glass Transition Temperature (Tg) of Latex Films

**Figure 4:**
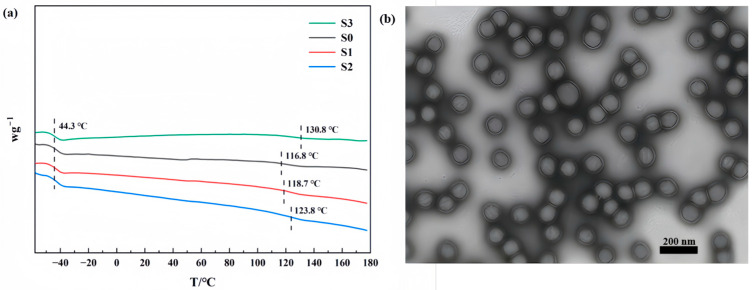
(**a**) Glass transition temperature profiles of polyacrylate latex films; (**b**) TEM image of latex particle morphology.

To evaluate the influence of DAAM content in ASR on the properties of corresponding latexes, the glass transition temperatures (Tg) of several latex films were measured by DSC, with the results shown in Figure 4a. As can be observed from Figure 4a, all four latexes were found to exhibit two distinct Tg values in their DSC curves, strongly demonstrating that the latex particles possess a typical core–shell structure due to the fact that the carboxylate ions on the ASR molecule are more hydrophilic than the intranuclear polymers, which form the shell layer of the ASR. Figure 4b directly visualizes the core–shell architecture of the latex particles via TEM. This result is consistent with previous findings from our research group, where BASF’s renowned industrial product J678 was used as a surfactant for the preparation of polyacrylate latexes [18].

At the same time, it is observed that Tg of polyacrylate latexes prepared with increasing amounts of DAAM on ASR tends to increase. In particular, when the amount of DAAM on the ASR reaches 3 wt%, the Tg of the shell layer of the corresponding latex S3 is 130.8 °C, which is significantly higher than the 116.8 °C of the shell layer of S0 without DAAM on the ASR. There are two possible reasons for this. On the one hand, DAAM polymers have a high Tg. According to the FOX equation, the Tg of a polymer is closely related to its composition. When the amount of DAAM is increased, the proportion of DAAM in the latex film increases, resulting in a change in the chemical composition of the overall latex film. In this case, the latex film is more likely to exhibit the high Tg characteristics of the DAAM polymer. On the other hand, DAAM and ADH undergo a ketohydrazide crosslinking reaction. This reaction results in the formation of a three-dimensional network structure in the latex films. In this structure, the movement of the molecular chain segments is hindered. The ability of the molecular chain segments to move is closely related to the Tg of the material, and when the movement of the molecular chain segments is impeded, a higher temperature is required to start the movement of the molecular chain segments, which means that the glass transition temperature is correspondingly increased. The three-dimensional network structure formed by the ketohydrazide crosslinking reaction makes the overall structure of the latex film more compact, greatly restricting the free movement of the molecular chain segments, which ultimately leads to an increase in Tg.

### 2.6. Water Contact Angle and Water Absorption of Latex Films

**Figure 5:**
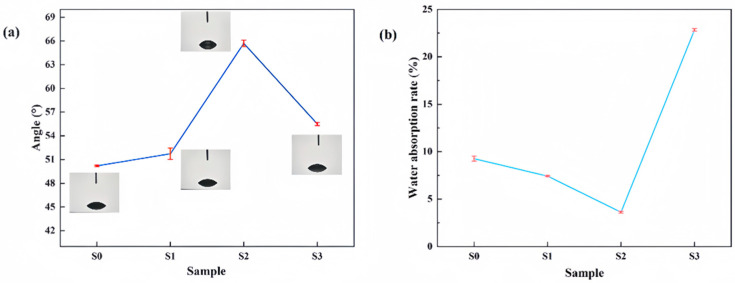
Water contact angle (**a**) and water absorption (**b**) of latex films.

The crosslinking density of latex films directly affects their water contact angle and water absorption rate. As shown in Figure 5, the water contact angles of the four acrylic latexes are S0, 50.2°; S1, 51.7°; S2, 65.7°; and S3, 55.5°. The corresponding water absorption are S0, 9.3%; S1, 7.4%; S2, 3.6%; and S3, 22.8%. It can be clearly observed that during the preparation of polyacrylate latex films from ASRs, the water contact angle of the latex films shows a tendency of increasing and then decreasing as the amount of DAAM used on the ASRs is gradually increased, whereas the water absorption rate, on the contrary, shows a tendency of decreasing and then increasing. At a low level of crosslinking within the system, a crosslinking network is built up between the molecular chains within the latex film as the ketohydrazide crosslinking reaction proceeds. The formation of this crosslinking network results in a relatively heterogeneous chain of molecules becoming regular and orderly, with a consequent reduction in the intermolecular gaps. This microstructural change directly leads to the surface of the latex film becoming more and more dense, thus making it difficult for water molecules to penetrate into the interior of the latex film. As a result, the water absorption rate tends to decrease during the initial phase when the crosslinking system is just introduced. At the same time, the water contact angle increases due to the increase in surface densification of the latex film. With the continuous introduction of crosslinking monomers, the crosslinked network of the latex film will reach an ideal state when a certain dose is reached. At this point, the interaction force between molecular chains becomes strong and can effectively prevent the penetration of water molecules. In addition, the formation of the crosslinked network will further change the surface energy of the latex film, thus increasing the hydrophobicity of the surface. The combination of these factors further leads to an increase in the water contact angle and a decrease in water absorption. However, when crosslinking is too high, the crosslinked network of the latex film becomes too tight and the flexibility of the latex film decreases drastically, which in turn leads to cracks and the formation of capillary channels, which leads to a drastic increase in water absorption. In addition, excessive crosslinking may also lead to an increase in the surface roughness of the latex film, resulting in a decrease in the water contact angle. It is noteworthy that when the DAAM content on ASRs reaches 2 wt%, the resulting polyacrylate latex films achieve an optimal balance between water contact angle and water absorption. At this critical dosage, the films exhibit a relative maximum contact angle (65.7°) and minimum water absorption (3.6%), demonstrating that precise control of crosslinking degree is essential for obtaining high-performance polyacrylate latex films.

### 2.7. Tensile Strength and Elongation at Break of Latex Films

**Figure 6:**
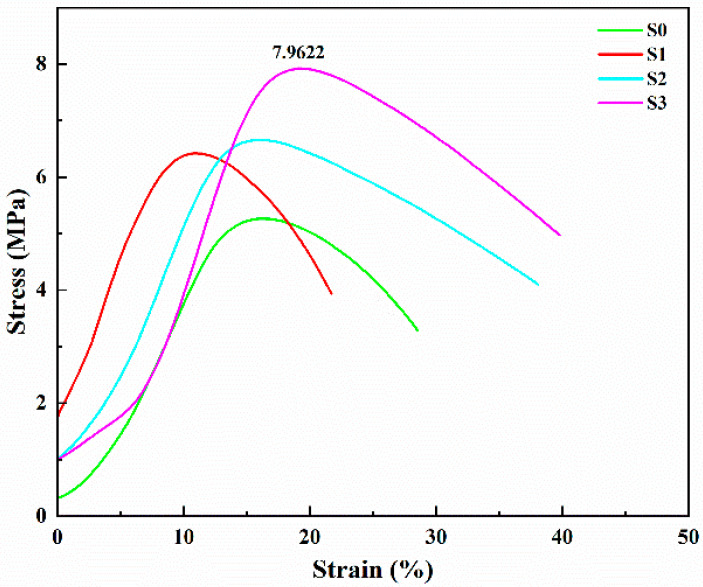
Tensile strength and elongation at break of polyacrylate latex films.

The tensile strength and elongation at break of the latex films were also measured in the experiment to investigate the effects of crosslinking on their mechanical properties. The results are presented in Figure 6. The tensile strength is 5.26 MPa for the latex S0 film, 6.42 MPa for the latex S1 film, 6.66 MPa for the latex S2 film, and 7.96 MPa for the latex S3 film, respectively. Clearly, the tensile strength of the polyacrylate latex films gradually increased with the increase in the amount of DAAM on ASR. The reason for this phenomenon is closely related to the introduction of a ketohydrazide crosslinking system in the polyacrylate shell layer. Ketohydrazide crosslinking, as an efficient means of crosslinking, has been shown to be effective in enhancing the tensile properties of polymers. Within the polyacrylate latex system comprising DAAM, the ketohydrazide crosslinking reaction occurs between the keto carbonyl group of the DAAM molecule and the hydrazide group of the ADH molecule. The chemical bonds formed through this crosslinking reaction have high strength and stability. It is these strong chemical bonds, which act as strong ‘connectors’ to link the polymer molecules together, that greatly enhance the intermolecular interaction forces and thus effectively increase the tensile strength of the polymer. This enables the latex film to better resist deformation and exhibit higher tensile strength when stretched by external forces.

The elongation at break of the four latex films was measured to be 28.56% (S0), 21.704% (S1), 38.11% (S2), and 39.78% (S3), generally showing a trend of increasing elongation with higher crosslinking density. This phenomenon can be explained as follows: Within a certain crosslinking range, the increase in DAAM content improves the distribution of crosslinking points, allowing the formation of a more effective network structure in the latex films. This results in a balance between strength and elasticity, leading to higher elongation at break in S2 and S3. The higher elongation of S0 (uncrosslinked) compared to S1 (low crosslinking) may be attributed to the disruption of physical entanglements between polymer chains by initial crosslinking. In low-crosslinking systems, the introduction of chemical crosslinks can interfere with the natural chain slippage mechanism, reducing elongation before an optimal network form.

### 2.8. Crosslinking Degree of Latex Films

**Figure 7:**
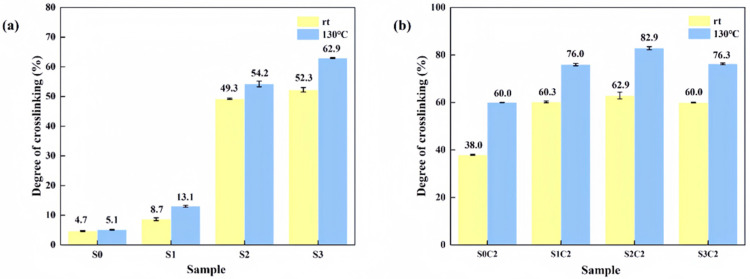
Crosslinking degree of latex films after treatment with different temperatures. (**a**) Crosslinking degree of latex films with shell-crosslinked structures after different heat treatments; (**b**) Crosslinking degree of latex films with core/shell-crosslinked structures after different heat treatments.

In the experiments, the crosslinking degree of the films formed from latexes S0, S1, S2, and S3 at different film-forming temperatures was first measured using the solvent extraction method. Figure 7a shows the crosslinking degrees of films formed at different temperatures: S0 films measured 4.7% at room temperature and 5.1% at 130 °C, S1 films showed 8.7% and 13.1%, S2 films exhibited 49.3% and 54.2%, while S3 films demonstrated 52.3% and 62.9% crosslinking degrees, respectively. The experimental data demonstrate that the crosslinking degree of latex films increases with both the DAAM content in ASRs and the film-forming temperature, which is consistent with theoretical expectations. The non-zero crosslinking degree observed for S0 (uncrosslinked control) may be attributed to chain transfer reactions between monomers during emulsion polymerization. It is particularly noteworthy that even when ASRs constitute only 30 wt% of the latex particles, the crosslinking density of the resulting films exceeds 30% when the DAAM content in ASRs reaches above 2 wt%. This phenomenon can be attributed to the formation of a highly crosslinked ASR shell structure within the latex particles, which effectively restricts the dissolution and migration of polymer molecules from the core layer. In addition, it was found that the degree of crosslinking of the 130 °C heat-treated latex film was overall higher than that of the room-temperature-formed film. This indicates that some functional groups are still present in the system that do not undergo crosslinking reactions during film formation at room temperature. In the heat treatment process, the molecular chain obtains enough energy, the diffusion ability is enhanced, and the number of effective collisions between molecules is significantly increased, which provides more favorable conditions for the crosslinking reaction, thus promoting more crosslinking reactions to occur and enhancing the crosslinking degree of the latex film.

Figure 7b shows the crosslinking degrees of latex films formed by four polyacrylate latexes (S0C2, S1C2, S2C2, and S3C2) at room temperature and 130 °C, respectively. These latexes were prepared by introducing DAAM (accounting for 2% of the core monomer mass) into the core layer of the latex particles. A comparison with Figure 7a reveals that the core–shell dual crosslinking system achieves a significantly higher crosslinking density than single-layer crosslinking. Latex films incorporating 2 wt% DAAM crosslinking monomer in the core layer demonstrate markedly enhanced crosslinking degrees compared to their counterparts without core-layer crosslinkers (S0, S1, S2, and S3). This phenomenon stems from the core’s substantial mass fraction (70% of the total particle), where the crosslinking of the core layer contributes more to the overall film crosslinking degree. By comparing S2 and S0C2, it is observed that S2 contains 2 wt% DAAM only in the shell layer, enabling crosslinking to occur exclusively in the shell, whereas S0C2 contains 2 wt% DAAM solely in the core layer, limiting crosslinking to the core. Although the amount of DAAM in S0C2 is 7/3 times that in S2—theoretically suggesting a higher crosslinking degree in S0C2—the actual crosslinking degrees of their latex films are nearly identical at both room temperature and 130 °C. This phenomenon indicates that the shell layer in S0C2 exhibits a strong shielding effect on the crosslinking reaction between DAAM in the core and the crosslinker ADH [18]. Furthermore, as shown in Figure 7b, the crosslinking degrees of the latex films formed by S2C2 at room temperature and 130 °C are 62.9% and 82.9%, respectively, while those of S3C2 are 60.0% and 76.3%. Despite S3C2 containing more DAAM in its shell layer and a higher overall DAAM content in the latex particles, its crosslinking degree is lower than that of S2C2. This can be attributed to the denser crosslinked network formed in the shell layer of S3C2, which imposes a greater hindrance to the crosslinking of DAAM in the core layer. These results indicate that although the core–shell dual crosslinking system is effective in improving the crosslinking degree of acrylate latex films, excessive crosslinking of the shell layer can severely hinder the crosslinking reaction of DAAM in the core layer. For the system studied here, the appropriate amount of DAAM crosslinker in the shell layer should be maintained at 2%.

### 2.9. Adhesion of Corresponding Water-Based Ink

**Figure 8:**
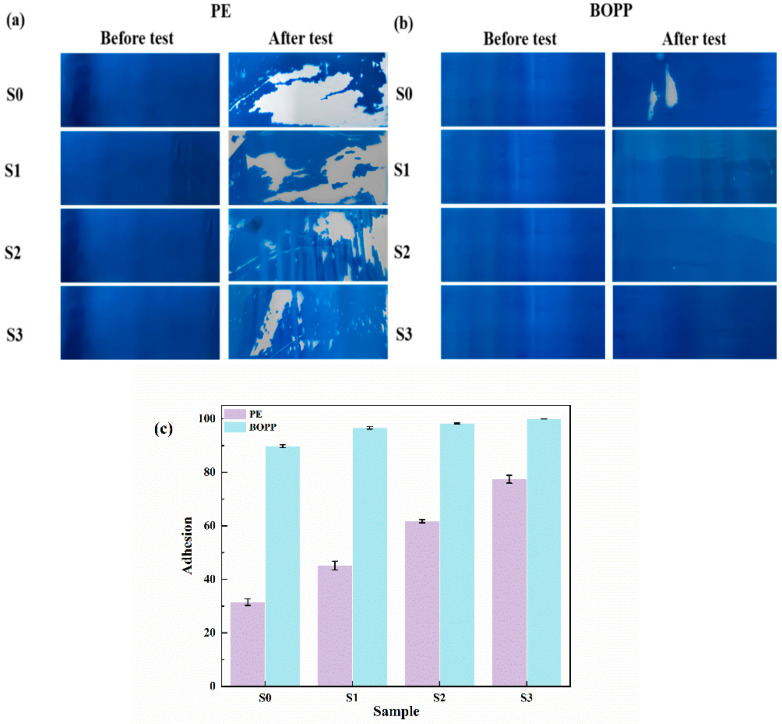
Adhesion of water-based inks: (**a**) photographic comparison of ink samples on PE substrates before and after adhesion testing, (**b**) photographic comparison of ink samples on BOPP substrates before and after adhesion testing, (**c**) adhesion of water-based inks made from the latex S0, S1, S2, and S3 on BOPP and PE substrates.

As clearly shown in Figure 8, with the increase in DAAM content in ASRs from 0 to 3 wt%, the adhesion of corresponding water-based inks on PE substrates progressively improved from 31.6% to 45.1%, then to 61.8%, and finally reached 77.4%, while on BOPP substrates, it increased from 89.8% to 96.7%, then to 98.4%, and ultimately achieved 100%, demonstrating DAAM’s positive contribution to adhesion enhancement. The improvement in adhesion with increasing ketone–hydrazide crosslinking degree can be primarily attributed to the following reasons. On one hand, as DAAM content increases, the crosslinking degree of the ketone–hydrazide system rises, enabling more chemical bonds between polymer chains and forming a tighter and more stable three-dimensional network structure. This structure not only enhances the internal cohesion of the water-based ink itself but also creates more physical or chemical bonding sites with the substrate surface. For instance, crosslinked molecular chains can better fill the microscopic voids on the substrate surface, increasing the contact area with the substrate and strengthening adhesion through mechanical interlocking. On the other hand, specific functional groups in DAAM may chemically react with the substrate surface, such as forming hydrogen bonds, covalent bonds, or other chemical linkages, or tightly combine with the substrate through physical interactions like van der Waals forces. As the crosslinking degree increases, more functional groups participate in interactions with the substrate, thereby significantly improving adhesion.

## 3. Materials and Methods

### 3.1. Materials

Styrene (St), methacrylic acid (MAA), butyl acrylate (BA), and methyl methacrylate (MMA), of industrial grade, were purchased from Shandong Ruifeng New Material Co., Ltd. (Zibo, China). Sodium dodecyl sulfate (SDS), of analytical grade, was purchased from Tianjin Fuchen Chemical Reagent Company Ltd. (Tianjin, China). Diacetone acrylamide (DAAM) and ADH, of analytical grade, were purchased from Shanghai McLean’s Biochemical Technology Co., Ltd. (Shanghai, China). Ammonia (25 wt%), of analytical grade, was purchased from Tianjin Fuchen Chemical Reagent Co., Ltd. (Tianjin, China). Sodium chloride, tert-butyl hydroperoxide (t-BuOH), and vitamin C, of analytical grade, were purchased from Shanghai Maclean’s Biochemical Science and Technology Co., Ltd. (Shanghai, China). Deionized water (DW), with an electrical conductivity ≤ 3.0 × 10^−5^ μs/cm, was self-made in our laboratory.

### 3.2. Method

#### 3.2.1. Synthesis of ASRs

The basic formulations of monomers for ASRs are listed in Table 3. First, appropriate amounts of deionized water, St, MMA, MAA, SDS, DAAM, and n-dodecyl mercaptan were added to a clean beaker according to the formulation and magnetically stirred for 30 min to prepare a pre-emulsion for later use. Next, certain amounts of deionized water and SDS were added to a four-neck flask. The reaction flask was heated to 80 °C, and under nitrogen protection, the initiator aqueous solution was added all at once. After stirring for 5 min, the pre-emulsion was added dropwise at a constant rate using a peristaltic pump for 40 min. After the dropwise addition was completed, the reaction was maintained at 80 °C with stirring for 60 min, yielding ASRs with an acid value of 130 mgKOH/g.

#### 3.2.2. Synthesis of Polyacrylate Latexes

The synthetic formulations of polyacrylate latexes using different ASRs were designed as shown in Table 4. First, appropriate amounts of deionized water, BA, and SDS were added to a clean beaker and vigorously stirred for 30 min to prepare the pre-emulsion, which was then set aside. Next, the pH of the ASR solution prepared in a four-neck flask was adjusted to approximately 8.5 using ammonia solution. Then, an initiator solution was added and the reaction vessel was heated to 80 °C, after which the pre-emulsion was added dropwise over 90 min. After complete addition, the reaction was allowed to proceed for an additional 2 h. Upon completion, the mixture was cooled to room temperature, followed by the addition of a specified amount of ADH and filtration to obtain the final polyacrylate latex with 40 wt% solid content.

In the experiments, the mass ratio of ASR to monomer was set at 30:70.

#### 3.2.3. Preparation of Latex Film

Approximately 10 g of latex was poured into a polytetrafluoroethylene (PTFE) mold and then dried either in an oven at 130 °C for 48 h or at room temperature (25 °C) for 7 days, yielding heat-cured latex films (130 °C) or ambient-cured latex films, respectively.

#### 3.2.4. Preparation of Water-Based Ink

Polyacrylate latex and color paste were added to the beaker in the ratio of 7:3, stirred at a certain stirring speed for one hour, dispersed homogeneously, and filtered to obtain water-based ink.

### 3.3. Characterization

The structure of latex film was measured using a Fourier transform infrared spectrometer (Tensor 37, Bruker Co., Ltd., Billerica, MA, USA). KBr tablets were used in sample preparation, and the wave number range of the test was 400–4000 cm^−1^. The molecular weight of the alkali-soluble resin and its distribution were determined by Waters gel permeation chromatography using tetrahydrofuran as the eluent. The particle size and zeta potential of the latex were tested by DLS measurement of particle size and distribution at 25 °C. DLS measurements were processed via Gaussian fitting, with parameters iteratively optimized using the Levenberg–Marquardt algorithm for nonlinear least-squares minimization, ensuring statistical robustness in particle size distribution analysis. A sample of the latex to be tested was diluted and placed in a cuvette three times and averaged. The zeta potential of the latex was then tested by selecting the zeta potential test mode and three measurements were averaged for each sample. Mechanical properties were tested with an electronic universal testing machine at 25 °C ambient temperature using prepared dumbbell-type latex film samples.

The latex solid content C% was determined gravimetrically by weighing 1–2 g of latex sample (m1) in an aluminum foil box (m0), then baking in an oven at 150 °C for 1.0 h. The sample was removed and weighed (m2) to calculate the solid content C% of the latex. After measuring the solid content C%, calculate the total feed volume of the emulsion polymerization as the total mass of the product Wt, the total mass of the monomers involved in the reaction is denoted as M, the total amount of non-volatile material in the raw material is denoted as Wu, and the polymer conversion rate T% is calculated.

(1) $$C\%=\frac{\left(m2-m0\right)}{m1}\times 100\%$$

(2) $$T\%=\frac{Wt\times C\%-Wu}{M}\times 100\%$$

A total of 5–10 mg of the latex film samples was taken and tested by differential scanning calorimetry (Differential Scanning Calorimeter DSC Mettler Toledo, Greifensee, Switzerland) to obtain the DSC curve, and then the Tg of the latex film was obtained.

The water contact angle of the prepared latex film samples was tested three times with a contact angle measuring instrument (water contact angle tester OCA25 Dataphysics, Filderstadt, Germany) in three different positions, at an ambient temperature of 25 °C, and the average value of the three tests was taken.

A small piece of the latex film sample was cut and weighed (denoted as ma). It was then immersed in deionized water at room temperature (25 °C) for 24 h. After removal, the surface moisture was dried, and the sample was weighed again (denoted as mb). The water absorption rate (W%) was calculated using the following formula:

(3) $$W\%=\frac{mb-ma}{ma}\times 100\%$$

The degree of crosslinking was tested by placing a w0 mass of latex film in a Soxhlet extractor and refluxing it in tetrahydrofuran solvent for 24 h. The final weight of the latex film is noted as w1 and the degree of crosslinking of the latex film is calculated as follows:

(4) $$crosslinking\%=\frac{w1}{w0}\times 100\%$$

Ink was applied to polyethylene (PE) and bi-oriented polypropylene (BOPP) substrates using an ink proofing machine and the substrates were dried in an oven at 60 °C for 10 min. Then, 3 M adhesive tape was applied to the PE or BOPP substrate, flattened 3 times, and quickly peeled vertically from the substrate surface.

## 4. Conclusions

This study first employed emulsion polymerization to synthesize crosslinkable ASRs with an acid value of 130 mgKOH/g, a number-average molecular weight of approximately 8500 Da, and DAAM crosslinking units incorporated into the resin molecular chains. The synthesized ASRs were then utilized as surfactants to prepare self-crosslinking polyacrylate latexes via an in situ one-pot emulsion polymerization approach. The resulting latexes exhibited excellent stability, with absolute ζ-potential values exceeding 30 mV and particle sizes around 100 nm. DSC analysis confirmed the core–shell structure of the polyacrylate latexes, with ASRs forming the shell layer. As the DAAM content in ASRs increased, the water absorption of latex films first decreased and then increased, while the water contact angle initially rose before declining, and tensile strength showed a gradual enhancement throughout. Crosslinking degree measurements revealed that introducing DAAM crosslinking systems simultaneously in both the shell and core layers effectively improved the film crosslinking degree. The core layer contributed more significantly due to its larger mass proportion in the latex particles. However, the crosslinked shell layer strongly shielded the core-layer DAAM’s crosslinking reaction. For latex S3C2 with 3 wt% DAAM in the shell, excessive shell crosslinking severely hindered core-layer crosslinking, resulting in a lower overall crosslinking degree compared to S2C2 containing 2 wt% shell-layer DAAM. The DAAM/ADH crosslinking system in ASRs significantly enhanced the adhesion of corresponding water-based inks on PE and BOPP substrates. When the amount of DAAM in ASR was increased from 0 to 3 wt%, its adhesion on PE increased from 31.5% to 77.4%, and its adhesion on BOPP even increased from 89.8% to 100%.

In conclusion, this work’s strategy of developing crosslinkable ASRs to enhance polyacrylate latex film crosslinking provides a novel approach for preparing high-performance polyacrylate latexes and their derived water-based inks.

## Figures and Tables

**Table 1:** Molecular weight and PDI of ASRs.

	Mn	Mw	MP	PDI
**ASR0**	8124	13,172	11,940	1.62
**ASR1**	8774	14,178	12,525	1.62
**ASR2**	10,370	18,503	14,506	1.78
**ASR3**	8565	13,277	11,757	1.55

**Table 2:** Conversion of polymerization reaction, solid content, and zeta potential of the latexes.

Latex Sample	S0	S1	S2	S3
Zeta/mV	−39.0	−41.7	−40.4	−43.1
Conversion/%	97.6	96.6	95.5	96.1
Solid content/%	39.0	38.6	38.2	38.4

**Table 3:** The basic formulations of monomers for ASRs.

ASR Code	St (g)	MMA (g)	MAA (g)	KPS (g)	SDS (g)	1-Dodecanethio (g)	DAAM (g)
ASR0	9	63	18	1.5	7.4	3.3	0
ASR1	9	63	18	1.5	7.4	3.3	0.9
ASR2	9	63	18	1.5	7.4	3.3	1.8
ASR3	9	63	18	1.5	7.4	3.3	2.7

**Table 4:** Synthetic formulations of latexes.

Latex Code	ASR (g)	NH_3_H_2_O (25%) (g)	NaCl (g)	KPS (g)	BA (g)	DAAM (g)
S0	ASR0/90	22	2.25	1.75	210	0
S1	ASR1/90	22	2.25	1.75	210	0
S2	ASR2/90	22	2.25	1.75	210	0
S3	ASR3/90	22	2.25	1.75	210	0
S0C2	ASR0/90	22	2.25	1.75	210	4.2
S1C2	ASR1/90	22	2.25	1.75	210	4.2
S2C2	ASR2/90	22	2.25	1.75	210	4.2
S3C2	ASR3/90	22	2.25	1.75	210	4.2

## Data Availability

The original contributions presented in this study are included in the article. Further inquiries can be directed to the corresponding author(s).
